# Effects of Fish Oil Supplementation during the Suckling Period on Auditory Neural Conduction in n-3 Fatty Acid-Deficient Rat Pups

**Published:** 2014-07

**Authors:** Vida Rahimi, Saeid Farahani, Atoosa Saeidpour, Shohre Jalaie, Parvane Mahdi

**Affiliations:** 1 *Department of Audiology , School of Rehabilitation, Tehran University ofMedical Science, Tehran, Iran.*; 2 *Department of Nutrition, School of Nutrition and Food Technology, Shahid Beheshti University of Medical Sciences, Tehran, Iran. *; 3 *Department of Statistics, School of Rehabilitation, Tehran University of Medical Sciences, Tehran, Iran. *

**Keywords:** Auditory Brainstem Response, Fish oil, n-3 Fatty Acid Deficiency, Rat pups.

## Abstract

**Introduction:**

Omega-3 fatty acid, particularly in the form of fish oil, has a structural and biological role in various systems of the body. The auditory and nervous systems are both influenced by omega-3 fatty acids, with omega-3 deficiency having devastating effects on both systems. Numerous studies have attempted to investigate this further. This study aimed to evaluate neural conduction in n-3 fatty acid-deﬁcient rat pups following supplementation with fish oil during the suckling period.

**Materials and Methods:**

In this interventional and experimental study, omega-3 fatty acids from fish oil (FO) were fed to rat pups of n-3 polyunsaturated fatty acid (PUFA)-deficient dams. Changes in auditory neural conduction were compared with those of control and n-3 PUFA-deficient groups using auditory brainstem response (ABR). The parameters of interest were P1, P3, and P4 absolute latency, P1-P3, P1-P4, and P3-P4 interpeak latency (IPL), and P4/P1 amplitude ratio. The rat pups were given oral FO, 5 ml/g weight for 17 days, between the age of 5 and 21 days.

**Results:**

No significant group differences were observed in P1 and P3 absolute latency (P>0.05), but there was a significant difference in P4 (P≤0.05). The n-3 PUFA-deficient + vehicle group had the most prolonged (the worst) P1–P4 IPL and P3–P4 IPL compared with control and n-3 PUFA-deficient + FO groups. There was no significant difference in P1–P4 IPL and P3–P4 IPL between the n-3 PUFA-deficient + FO group and the control group (P>0.05). There was a significant effect of diet on P1–P4 IPL and P3–P4 IPL between groups (P≤0.05).

**Conclusion:**

The results of present study showed the effect of omega-3 deficiency on auditory neural structure during pregnancy and the lactation period. Additionally, we observed reduced reduction in devastating effects on neural conduction in n-3 fatty acid-deﬁcient rat pups following the supplementation of FO during the suckling period

## Introduction

Omega-3 and omega-6 essential fatty acids (FAs) have an important role in structural, functional, as well as in different biological aspects in the body (1,2). Essential FAs are not manufactured in the human body but need to be provided through the consumption of certain food (1–3). The biological effects of omega-3 and omega-6 FAs, which are characterized by mutual interactions, have a strong influence on multiple functions in the human body (3). Omega-3 FAs, especially docosahexaenoic acid (DHA; 22:6n-3) and eicosapentaenoic acid (EPA; 20:5n-3), are responsible for several vital body functions including the development of the nervous system (4-6). DHA also acts a ligand for the retinoid receptor (the main step in the regulation of gene expression) in neural tissue (5). Additionally, the role of omega-3 in neural myelination cannot be ruled out **(**5**,**^6^**). **According to some studies, the use of dietary supplements during pregnancy and lactation can have an impact on fetal and infant development (4). 

The effect of n-3 FA deficiency in the pre- and postnatal period causes decreased brain weight, altered nerve FA composition, impaired visual function, neural structural malformation, a more rapidly aging brain in old age, and impaired auditory nerve conduction, as demonstrated by abnormal auditory brainstem response (ABR) (7,8).

Recent studies have been revealed that DHA deficiency in the brain, retina, liver, and kidney is caused by n-3 polyunsaturated fatty acids (PUFA) deficient feeding over two generations, although the deficiency can be recovered within eight weeks after initiation of a repletion diet (9,10).

It is known that ABR is an important tool for measuring sensory and functional development of the central auditory nervous system, and this test is therefore included in most animal studies in the auditory field **(**11**,**^12^**).** Additionally, ABR is a sensitive tool for detecting the effects of diet on the development of sensory and nervous system up to the inferior colliculus (5). Since the brainstem neural transmission time gives an initial estimate of the degree of neural myelination, and because dietary fat may be associated with myelin repair, ABR can be used as a tool to assess the effects of omega-3 on the growth of neurons during infancy (11,7).

To date, no report has been published related to the effect of DHA deficiency on the auditory system. This prompted us to undertake the current investigation, in which we examined the effect of an n-3 PUFA-deficient nutritional status on auditory neural conduction time. As the status of severe n-3 PUFA deficiency is so rare in humans, we included cases with a mild deficiency only. Furthermore, we investigated the effect of fish oil (omega-3) supplementation during the suckling period on auditory variables in n-3 fatty acid-deficient rat pups by comparing the results of ABR in n-3 PUFA-deficient rat pups who were supplied with fish oil compared with no fish oil supplementation. 

The results of such a study may provide a route to improving auditory neural conduction in n-3 PUFA deficiency through the supplementation of fish oil during the suckling period.

## Materials and Methods

All animal procedures were performed according to the Regulations for Animal Experiments in Tehran University of Medical Sciences and with ethical approval (project No 91-01-32-17279) from the ethical committee. 

*Animal, Diets, and Supplementation*

Female Wistar rats, 10 weeks of age, were purchased from the Pasteur Institute (Tehran, Iran). They were kept for a week to comply with condition. They were mated individually with male Wistar rats in suspended cages. The introduction of a sperm plug was designated as gestational Day 1. The females were then placed in separate polycarbonate cages. The 20 females were randomly assigned to one of the two diet conditions starting from Day 1 of pregnancy. The two diets were formulated according to AIN-93G standards as a control diet and the n-3 PUFA-deficient diet by Dyets Inc (Bethlehem, PA,USA) and Damloran Razak Inc (Borojerd, Iran). The detailed composition of each diet is given in (Table 1).

**Table 1:** Composition of the diets (g/kg).

**Nutritional Substance**	**Control**	**n-3 PUFA** **deficient**
Casein	200	200
Cornstarch	397.486	397.486
Dextrinized cornstarch	132	132
Sucrose	100	100
Cellulose	50	50
peanut and safflower oils	-	70
Soybean oil	70	-
Vitamin mix	10	10
Mineral mix	35	35
L-cystine	3	3
Choline bitartrate	2.5	2.5
tert-Butylhydroquinone	0.014	0.014
Calories (kcal/g)	3.96 kcal/g.	3.96 kcal/g.

The control diet based on soybean oil, whereas the n-3 PUFA-deficient diet contained a mixture of peanut and safflower oils in place of soybean oil. The n3/n6 ratio in soybean oil in the control group was 0.012 and 0.002 for the n-3 PUFA-deficient oil. 

Menhaden oil supplement (Dyets Inc, Bethlehem, PA, USA) was used as a source of n-3 LCPUFA. The fatty acid composition of the three oils is given in (Table. 2). 

The menhaden oil was emulsified in 1% sodium carboxymethyl cellulose (CELLO GEN F, Japan) solution by sonication to a final concentration of 20% (w/w).The emulsions were freshly prepared daily. 

**Table 2:** Fatty acid composition of the three oils used in the study (% of total fatty acids).

**Fatty acid**	**Soybean oil**	**peanut + safflower oils**	**Menhaden fish oil supplement**
12:0	-	-	0.11
14:0	0.07	0.03	7.96
16:0	10.97	6.8	19.88
17:0	-	-	1.17
18:0	4.17	2.2	1.99
20:0	0.35	-	0.61
22:0	0.36	-	0.32
24:0	-	-	0.20
16:1 (n-7)	-	-	10.69
17:1 (n-8)	-	-	2.24
18:1 (n-9)	25.85	67.8	16.38
22:1 (n-9)	0.01	-	0.23
17:4	-	-	3.91
18:2 (n-6)	51.40	22.4	2.26
20:3 (n-6)	-	-	0.38
20:4 (n-6)	-	-	0.20
18:3 (n-3)	6.34	0.06	2.04
18:4 (n-3)	-	-	3.68
20:5 (n-3)	-	-	1.90
22:5 (n-3)	-	-	2.53
22:6 (n-3)	-	-	14.68
n3/n6	0.12	0.002	11.04

*Experimental Procedure*

Dams (n=6 per group) were fed the experimental diets throughout the gestation and lactation periods. After parturition, male pups in the n-3 PUFA-deficient diet group were divided into two groups. Pups aged 5–21 (n=6) in one of these groups were orally administered fatty acid supplements at 5 ml / g of body weight. Other male pups on the n-3 PUFA-deficient diet (n=6) and the control diet were administered the vehicle (n=6). The fish oil (FO) or vehicle administration continued for 17 days, ending on the weaning day. After weaning, pups were fed the same diet as their dams for a week without oil or vehicle administration. After a 1-week supplement-free period (28 days of age), all pups underwent an ABR test.

*ABR Procedure*

Prior to ABR recording, animals were anesthetized with a mixture of xylazine (10 mg/kg) and ketamine (40 mg/kg) admin- istered intramuscularly. The temperature was monitored because temperature can influence the ABR results. The limits of temperature were ±1° (13). A water-circulating heating pad was used to regulate and maintain normothermia by raising or lowering the temperature of the circulating water.

The ABR was differentially recorded between two subcutaneous platinum E-2 needle electrodes. An inverting needle electrode was subcutaneously placed below the test ear as well as a non-inverting electrode at the vertex. A ground electrode was also positioned below the contralateral ear. Evoked potentials were collected by a Biologic Navigator (Natus, USA) and amplified by a factor of 300,000 times with a digital bandpass of 100–3000 Hz. Electrode impedances ranged from 0–5 kΩ. At least 256 responses were averaged. 

The amplified signals were averaged with positivity displayed upwards and traces stored on computer disk for a later analysis. Recordings were made in an electrically shielded sound box (30×60×30 cm). Calibration intensity level in the sound box was performed with a Norsonic sound level meter (Norsonic, Norway) (1/3 octave, impulse and peak state). The speaker’s intensity level at a distance of 10 cm from the ears of the rat was measured according to the level of output in the ABR instrument and was calibrated according to ANSI standard. Click stimuli were delivered through a high-frequency loudspeaker (HD250) with a flat frequency response plot to 20 KHz positioned directly in front of the animal in 100 dB peSPL (duration = 100 μs, polarity = refraction, repetition rate=11.1/s). 

The analysis epoch was 10.24 ms. An artifact rejection system eliminated indi- vidual responses if they displayed intensity exceeding 47.3 SPL. Two ABR traces were collected in 100 dB peSPL level intensity.

The ABR is composed of four vertex-positive peak waves (P1 to P4) within 6 ms of stimulus onset (14). These peaks reflect neural activity chiefly from the auditory nerve (P1), the cochlear nucleus (P2), the superior olivary complex (P3), and the lateral lemniscus and/or inferior colliculus(IC) (P4) (15,16). The primary outcome variables were the P1, P3, and P4 absolute latency, the measure that was the indicator of cochlear and neural transmission time along the auditory nerve and brainstem auditory pathway, in response to the 100 peSPL stimuli. The secondary outcomes were P1-P3, P3-P4, and P1-P4 interpeak latency (IPL). The P1- P3 IPL is assumed to measure neural transmission from the auditory nerve to the superior olivary complex. The P1–P4 IPL measures the brainstem portion of neural transmission by excluding the auditory nerve transmission time, and P3–P4 IPL measures neural transmission in the upper brainstem area (16). The third outcome variable was P4/P1amplitude ratio that was an indicator of magnitude of response.

*Statistical Analysis*

All data are expressed as the means ± standard deviation (SD). Due to the limited size of the sample, a Kruskal-Wallis nonparametric test was used to determine whether the values of variables in n-3 PUFA-deficient + FO, n-3 PUFA-deficient + vehicle, and control groups differed from each other. A Mann-Whitney U test was used for comparisons between pairs of groups. In all instances, the criterion for statistical significance was defined as P≤0.05. IBM SPSS Statistic ver. 20 (IBM Corp, USA) was used for all statistical calculations.

## Results

*Absolute Latency (Neural Transmission Times)*

There were no significant group differences in P1 and P3 absolute latency P=0.80, P=0.65, respectively. However the Kruskal-Wallis non-parametric test showed significant differences in P4 absolute latency between groups (P=0.003). Statistical analysis showed the most significant (worst) increase in P4 absolute latency in the n-3 PUFA-deficient + vehicle group compared with control and n-3 PUFA-deficient + FO groups (P≤0.05). A lower (improved) P4 absolute latency in the n-3 PUFA-deficient + FO groups was shown in comparison with n-3 PUFA-deficient + vehicle mice (P≤ 0.05) (Table. 3). No significant difference was observed in P4 absolute latency between the control and n-3 PUFA-deficient+FO groups (P>0.05) (Table. 3). 

**Table 3:** ABR Absolute latency (ms) as a function of Diet Group (mean±SD).

Absolute latency	Control	n-3 PUFA deficient + vehicle	n-3 PUFA deficient + FO	P value
P1	1.53±0.12	1.57±0.12	1.52±0.10	N.S. (0.80)
P3	3.35±0.14	3.41±0.12	3.35±0.19	N.S. (0.65)
P4	4.61±0.07	4.96±0.13^a^^b^	4.57±0.14	0.003

N.S. = not significant.

a Significantly different from control group, P=0.004

b Significantly different from n-3 PUFA-deficient + FO group, P=0.004

*Interpeak Latency*

There were no significant group differences in P1-P3 IPL as a function of diet (P>0.05). There was a significant effect of diet on P1-P4 IPL and P3-P4 IPL (P≤0.05). The n-3 PUFA-deficient + vehicle had the most prolonged (worst) P1-P4 IPL and P3-P4 IPL compared with the control and n-3 PUFA-deficient + FO groups. There was no significant difference in P1-P4 IPL and P3-P4 IPL between the n-3 PUFA-deficient + FO and control groups (P>0.05). The P1-P4 IPL and P3-P4 IPL in the n-3 PUFA-deficient + FO groups were significantly shorter than the n-3 PUFA-deficient + vehicle group (P≤0.05) (Table. 4). 

**Table 4:** ABR Interpeak latency (ms) as a function of Diet Group (mean±SD).

Interpeak latency	Control	n-3 PUFA deficient + vehicle	n-3 PUFA deficient + FO	P-value
P1-P3	1.84±0.24	1.84±0.18	1.82±0.25	N.S.(0.99)
P3-P4	1.32±0.11	1.53±0.12^ab^	1.21±0.11	0.01
P1-P4	3.11±0.20	3.37±0.22^ ab^	3.05±0.15	0.048

*P4/P1 Amplitude Ratio*

The mean values of the P4/P1 amplitude ratio in the control (0.88±0.49), n-3 PUFA-deficient + vehicle (0.73±0.54), and n-3 PUFA - deficient + FO group (0.78±0.64) showed no significant differences (P>0.05).

## Discussion

Church et al demonstrated that the amount of omega3 and omega6 feeding formula had a significant effect on IC serotonin (16,17). A low concentration of omega3 in diets leads to decreased monoamine neurot- ransmitter concentrations in different brain regions, especially IC(the origin of P4) (18). Consequently, we would expect an increase in P4 latency. Bourre et al (1999) showed the effect of alpha-linolenic acid deficiency only on delayed P3 latency (19). These finding were in line with our results. We found that a mild deficit of n-3 FA in the maternal diet during pregnancy and lactation caused prolonged auditory neural conduction in rat pups at 28 days of age. These effects were only significant at P4 absolute latency. 

This prolonged ABR latency was an indicator of delayed neural transmission times due to poor neuronal myelination or poor synaptic growth along the brainstem portions of the auditory pathway.

Our investigation showed a decrease in P4 latency after taking FO supplements in rat pups compare with an n-3 PUFA-deficient + vehicle group. Reduction in P4 latency could be due to the recovery in neural pathways myelin. One study also showed that adequate amounts of DHA and arachidonic acid (AA; 20:4 n-6) can lead to increased levels of dopamine and improved performance in IC (16). This finding is likely to be the reason for the reduction of P4 latency in our present study.

Considering IPL, our results showed P1-P4 IPL and P3-P4 IPL delay in the deficient group with no impact on P1-P3 IPL. Prolonged P4 absolute latencies without any increase in P1-P3 IPL lead to this finding, and was the indicator of impaired neuronal function in the anatomical region between peaks 3 and 4, which is the upper area in brainstem (14). The results of Church et al (2008, 2010), also confirm our results (16,17). 

Additionally, our investigations showed a decrease in P1-P4 IPL latency after FO supplements were taken by pup rats. After taking FO during the suckling period, the result of the P1-P4 IPL latency did show have a significant difference when compared with the latency of the normal group, indicating that the neural deficiency compensated for latency. 

In previous studies, the P3-P4 IPL was not used, but this variable was examined in our project to determine the location of lesions in the upper level of the brainstem. 

There was no significant difference between the groups in the P4/P1 amplitude ratio. None of the previous studies evaluated the P4/P1 amplitude ratio. We had expected that the neurologic deficit would affect the P4/P1 amplitude ratio due to the involvement of the nerve fiber (14). 

It may be preferable in future studies to use P2 (the maximum amplitude) to evaluate amplitude. 

## Conclusion

The results of the present study showed the effect of omega3 deficiency on auditory development in pregnancy and the lactation period and compensation in neural conduction in n-3 fatty acid-deﬁcient rat pups following supplementation of FO consumption during the suckling period.
